# Epigenetic Hierarchies in Autism from Folate–SAM Metabolism to LSD1 and G9a

**DOI:** 10.3390/biom16101411

**Published:** 2026-09-28

**Authors:** Nuria Pérez, Aina Bellver, Fahad Hussain, Elena Huerta-Ramos, Nuria Tous, Richard Eugene Frye, Christian Griñán-Ferré

**Affiliations:** 1 Department of Pharmacology, Toxicology and Therapeutic Chemistry, University of Barcelona, 08028 Barcelona, Spain; nperezsa21@alumnes.ub.edu (N.P.); abellversanchis@ub.edu (A.B.); fahadhussain1077@gmail.com (F.H.); 2 Institute of Neurosciences of the University of Barcelona, University of Barcelona, 08035 Barcelona, Spain; 3 Hospital de Dia Numancia, Parc Sanitari Sant Joan de Déu, 088029 Barcelona, Spain; mariaelena.huerta@sjd.es (E.H.-R.); nuria.tous@sjd.es (N.T.); 4 Institut de Recerca Sant Joan de Déu (IRSJD), Etiopatogènia i Tractament dels Trastorns Mentals Greus (MERITT), 08830 Sant Boi de Llobregat, Spain; 5 Biomedical Research in the Mental Health Network (CIBERSAM), Carlos III Health Institute, 28029 Madrid, Spain; 6 Autism Discovery and Treatment Foundation, Phoenix, AZ 85050, USA; drfrye@autismdiscovery.org; 7 Rossignol Medical Center, Phoenix, AZ 85050, USA; 8 Centro de Investigación en Red, Enfermedades Neurodegenerativas (CIBERNED), Instituto de Salud Carlos III, 28031 Madrid, Spain

**Keywords:** autism spectrum disorder, epigenetic hierarchy, hierarchical prioritization, folate metabolism, one-carbon metabolism, SAM-dependent methylation, histone methylation hierarchy, H3K4me2, H3K9me2, LSD1, G9a

## Abstract

Autism spectrum disorder (ASD) is a diverse neurodevelopmental condition involving interactions among genetic, environmental, metabolic, immune, synaptic, and epigenetic factors. This review explores whether disruptions in folate-dependent one-carbon metabolism and S-adenosylmethionine (SAM) availability may increase epigenetic susceptibility in specific ASD subgroups based on biological criteria. This narrative, hypothesis-driven review synthesizes selected peer-reviewed literature encompassing human studies, animal models, cell systems, and biochemical research. Findings including decreased methylation capacity, altered DNA methylation, and abnormal histone methylation have been reported in ASD and related neurodevelopmental conditions. Nonetheless, evidence strength varies considerably depending on phenotype, tissue type, developmental stage, and experimental system. On this basis, a provisional SAM-dependent epigenetic hierarchy is proposed in which methyltransferases may differ in vulnerability to metabolic stress according to enzyme kinetics, subcellular context, developmental timing, and compensatory regulation. This model is conceptual rather than an established mechanism demonstrated uniformly across ASD. Clinical and translational findings are interpreted cautiously. Evidence for folinic acid is strongest in selected subgroups such as folate receptor alpha autoantibody-positive ASD and cerebral folate deficiency-related presentations, whereas LSD1/KDM1A and G9a/EHMT1/2 modulation remains largely preclinical. Overall, the review argues that folate–SAM-related epigenetic dysregulation may represent one contributory pathway in some ASD subgroups and provides a framework for future mechanistic and stratified therapeutic studies.

## 1. Convergent Molecular Pathways and Metabolic Foundations in Autism Spectrum Disorder

Neuroinflammation, oxidative stress (OS), mitochondrial dysfunction, and synaptic dysregulation are distinct molecular processes, but they are deeply linked through epigenetic regulation [1]. These ‘convergent pathways’ both arise from and contribute to epigenetic dysregulation: neuroinflammatory cytokines promote G9a-mediated H3K9me2 accumulation; mitochondrial dysfunction affects SAM compartmentalization; OS causes epigenetic changes through non-enzymatic histone modifications. This feedback loop explains why targeting a single pathway often yields limited success, whereas focusing on the epigenetic core offers a more comprehensive solution.

Unlike previous reviews that examined folate-dependent one-carbon metabolism and epigenetic dysregulation in ASD as separate areas, this review introduces a novel integrated, tiered SAM-dependent methyltransferase vulnerability model. This model connects folate-pathway genetics, SAM/SAH biochemistry, and chromatin-modifying enzyme pharmacology (LSD1/KDM1A and G9a/EHMT2) within a hierarchical framework. Additionally, it explicitly stratifies clinical and translational implications based on ASD biomarker subgroups, rather than viewing ASD as a uniform epigenetic condition.

### Introduction: Autism Spectrum Disorder, Convergent Mechanisms, and Rationale for a Folate–SAM Framework

Autism spectrum disorder (ASD) is a heterogeneous neurodevelopmental condition characterized by differences in social communication and restricted or repetitive patterns of behavior, with marked biological and clinical variability across affected individuals [2]. Current evidence supports contributions from genetic, synaptic, immune, metabolic, environmental, developmental, and epigenetic mechanisms rather than a single unifying cause [3].

According to data from the CDC’s Autism and Developmental Disabilities Monitoring (ADDM) network, the estimated prevalence of ASD among 8-year-old children in the United States was 1 in 31 in 2022, representing a continued increase in prevalence over time. Among 4-year-old children, the estimated prevalence was approximately 1 in 34, corresponding to 0.9 times the prevalence observed among 8-year-old children [4]. In contrast, epidemiological data on the prevalence of ASD among adults remain limited. A CDC study estimated that approximately 2.21% of the U.S. population aged 18 years and older were living with ASD in 2017 [5].

This review should be viewed as a narrative, hypothesis-driven overview. It does not aim to present the comprehensive methodology typical of a systematic review or meta-analysis. Instead, it synthesizes selected evidence from human studies, post-mortem analyses, peripheral biomarker research, animal models, cell systems, and biochemical investigations to assess whether folate–SAM metabolism could be a mechanistically relevant pathway in certain ASD subgroups.

To improve source-to-claim traceability, the levels of evidence used throughout the review are distinguished as follows: (i) direct human evidence, including cohort, cerebrospinal fluid (CSF), peripheral blood, and post-mortem brain studies; (ii) indirect mechanistic evidence from animal models and patient-derived or engineered cell systems; (iii) biochemical and in vitro enzymology informing methyltransferase properties; and (iv) integrative hypotheses proposed here to connect these observations into a SAM-dependent epigenetic framework. Where the available literature is associative, this is described as such and should not be interpreted as proof of causality.

Within this framework, the review aims not to assert that ASD is solely or uniformly epigenetic but to explore whether folate-dependent methylation and chromatin regulation could account for some converging neurobiological findings in ASD and lead to testable hypotheses. Therapeutic implications are considered cautiously, clearly separating clinical observations in specific subgroups from mostly preclinical data on chromatin-modifying enzymes. Recent evidence has shifted the view of ASD from being a solely behavioral disorder to a condition with distinct biological foundations, marked by multiple interconnected physiological abnormalities [6,7]. Although these mechanistic pathways originate from different sources, they ultimately converge on shared cellular and molecular dysfunctions that collectively shape the ASD phenotype. Neuroinflammation and immune dysregulation are key components of this process. Analyses of CSF show increased levels of various inflammatory cytokines, such as TNF-α, IL-4, IL-21, BAFF, IL-31, IL-5, and APRIL [8], and brain studies confirm that these inflammatory markers are not just peripheral but indicate genuine central nervous system inflammation, with higher levels of TNF-α, IL-6, IFN-γ, and IL-8 found in post-mortem brain samples from individuals with ASD [9,10]. This inflammatory process disrupts blood–brain barrier integrity, activates microglia, and alters neurotransmitter systems, creating a vicious cycle that worsens neuronal dysfunction [8]. The immune dysregulation in ASD extends to both innate and adaptive immunity, with T cell populations showing abnormal helper/suppressor ratios, decreased numbers of CD4+ and CD8+ T cells producing IFN-γ and IL-2, and a dysregulated Th1/Th2 balance [9,11]. B cell abnormalities include elevated expression of CD23+ B lymphocytes and altered immunoglobulin profiles, with some studies reporting reduced IgG and IgM levels while others describe increased food-specific IgA, IgG, and IgE antibodies [9]. Notably, the severity of behavioral symptoms in ASD correlates directly with the degree of immune dysregulation, as higher levels of pro-inflammatory cytokines (IL-6, IL-1β, TNF-α, IL-17A) are associated with more severe social impairments, communication deficits, and repetitive behaviors [9,11].

Within this pro-inflammatory milieu, OS and redox imbalance emerge as critical pathophysiological mechanisms that further compromise neuronal function [11,12]. In ASD, OS is marked by an imbalance between reactive oxygen species (ROS) production and antioxidant defenses, with analyses of post-mortem brain tissues revealing notably lower levels of glutathione (GSH), the primary cellular antioxidant, especially in the cerebellum and Brodmann Area 22 (BA22) [12,13]. At the same time, indicators of oxidative damage are significantly increased: 3-nitrotyrosine (3NT), which reflects protein oxidation, and 8-oxo-deoxyguanosine, a marker for DNA oxidation, are elevated across various brain regions, including the cerebellum, temporal cortex, and prefrontal cortex [12,13]. The GSH/GSSG (oxidized glutathione) ratio, a key marker of cellular redox balance, is significantly reduced in ASD brain tissue and correlates with disease severity [12]. Notably, the regions exhibiting the highest OS, such as the prefrontal cortex, temporal cortex, and cerebellum, align with areas involved in speech, social behaviors, and sensory–motor coordination [13]. Additionally, antioxidant enzyme systems attempt to respond adaptively but remain insufficient, as evidenced by decreased superoxide dismutase 2 (SOD2) activity in temporal lobe samples from children with ASD [14]. Mitochondrial dysfunction provides an additional layer of convergence between OS, neuroinflammation, and altered energy metabolism in ASD. Research suggests that 5–30% of people with ASD show signs of mitochondrial dysfunction, with some groups experiencing severe bioenergetic issues [15,16]. Post-mortem brain studies identify deficiencies in the electron transport chain (ETC) complexes, mainly Complex I and Complex III, in the temporal and frontal cortex [14,17]. These ETC abnormalities lead to decreased efficiency in oxidative phosphorylation (OXPHOS), reduced ATP production, and unexpectedly higher ROS levels, thereby sustaining a self-perpetuating cycle of oxidative damage [15,17]. Consistent with these findings, lymphoblastoid cell lines (LCLs) from children with ASD show abnormal mitochondrial respiration patterns, with some displaying significantly higher oxygen consumption rates (OCRs) in all bioenergetic measures, such as ATP-linked respiration, proton leak, and maximum respiratory capacity [18]. Disrupted mitochondrial calcium (Ca^2+^) cycling further contributes to synaptic dysfunction, as abnormal Ca^2+^ release can activate microglia, trigger neuroinflammation, and promote programmed cell death, [15] while metabolomic analyses indicate changes in tryptophan metabolism and impaired NAD+ production routes in ASD, further affecting mitochondrial energy function [18].

At the synaptic level, many of these upstream alterations culminate in synaptic dysfunction and an imbalance between excitatory glutamatergic and inhibitory GABAergic neurotransmission (E/I imbalance) [19,20]. Evidence supporting this hypothesis includes the observation that 10–30% of people with ASD experience epilepsy, indicating significant disruptions in inhibitory signaling [20]. Neurochemical studies show decreased GABA levels and altered glutamate concentrations, resulting in a low glutamate/GABA ratio that interferes with normal synaptic balance [19]. The molecular basis for this E/I imbalance involves mutations in key synaptic adhesion molecules and scaffolding proteins. Genes such as neurexins (NRXN1, NRXN2) and neuroligins (NLGN3, NLGN4), which form trans-synaptic complexes essential for synapse formation and function, are among the most commonly mutated in ASD [21,22]. Disruptions in postsynaptic density proteins like PSD-95 (encoded by DLG4) and SHANK proteins impair synaptic connectivity and plasticity [23]. Additionally, brain-derived neurotrophic factor (BDNF), which supports the development and maturation of both glutamatergic and GABAergic synapses during postnatal development, is significantly reduced in ASD models [23]. BDNF deficiency during critical developmental windows causes a progressive E/I imbalance, starting with deficits in excitatory input that eventually lead to both excitatory and inhibitory dysfunctions [23].

**Figure 1:**
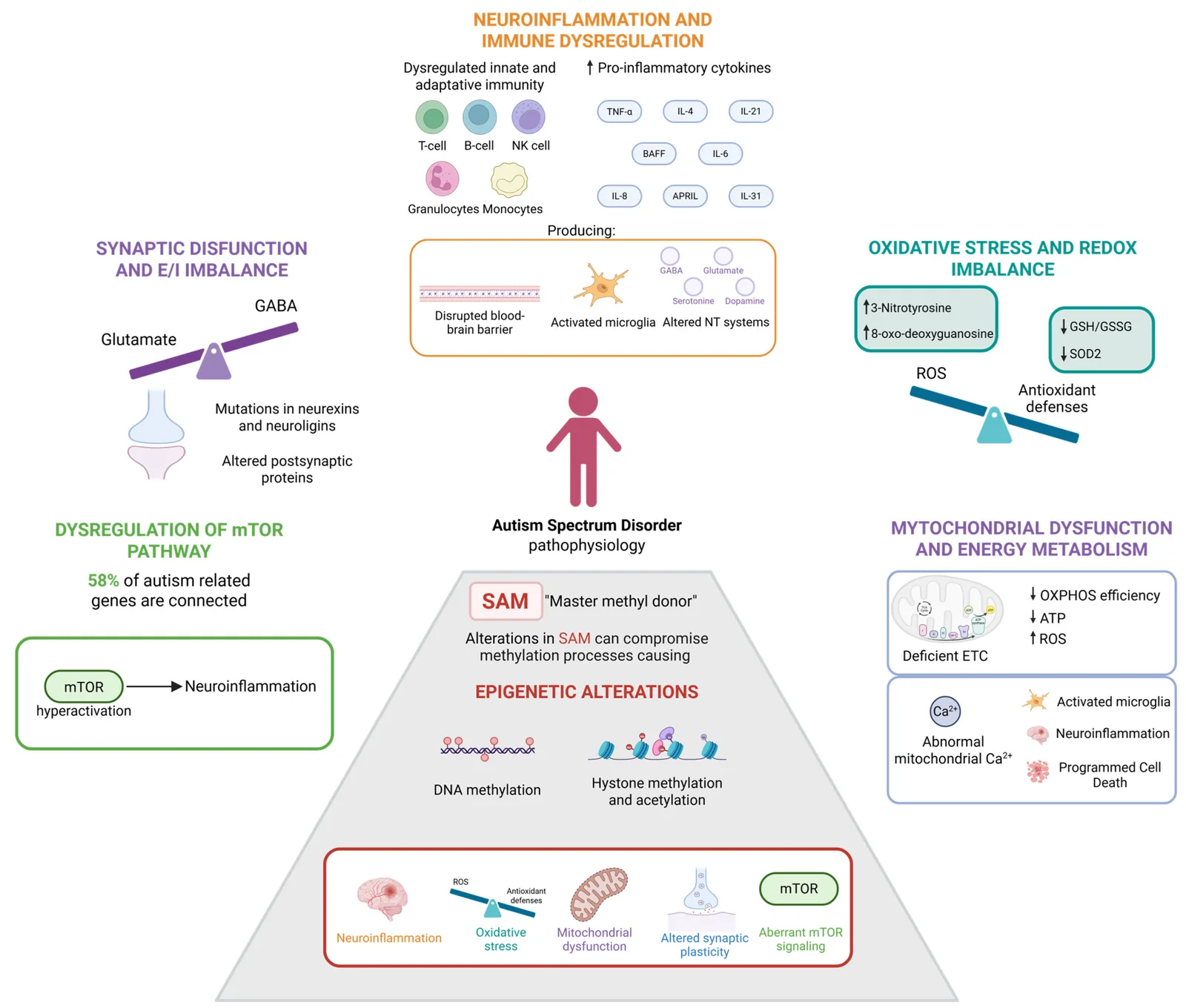
Overview of molecular pathways implicated in ASD, focusing on epigenetic alterations and the effects of SAM levels. This schematic frames the working hypothesis that folate–SAM metabolism constitutes a convergence point for these upstream pathways, explored mechanistically in subsequent sections.

Finally, dysregulation of the mechanistic target of rapamycin (mTOR) signaling pathway provides a unifying framework that links several of these molecular disturbances. About 8–10% of ASD cases show abnormalities in the mTOR pathway, and up to 58% of autism-related genes are connected to mTOR signaling, either directly or indirectly [24,25]. Mutations in negative regulators of mTORC1, such as TSC1, TSC2, PTEN, and NF1, cause mTOR hyperactivation, leading to ASD-like features including megalencephaly, social deficits, and repetitive behaviors. The mTOR pathway controls vital neuronal functions, including protein production at dendritic spines, synaptic plasticity, autophagy, and neuronal migration [24,25]. Abnormal mTOR signaling results in excessive protein synthesis, disrupted cortical connections, changes in dendritic spine density and shape, and faulty synaptic pruning [24]. Recent unbiased phospho-proteomics research shows that genetically different ASD subtypes share similar mTOR pathway changes, indicating this as a common disease mechanism [26]. Additionally, mTOR dysregulation influences neuroinflammation by controlling cytokine production from microglia and other immune cells, with overactive mTOR linked to higher CCL5 levels and ongoing neuro-inflammatory responses (Figure 1) [27].

## 2. Folate–Epigenetic Pathways and Integration with Core Disease Mechanisms

The link between epigenetics and folate metabolism has become a key focus in ASD research [28,29]. It provides essential insights into disease mechanisms and opens up potential treatment avenues. This pathway highlights how folate (vitamin B9) influences thousands of genes by functioning as an essential cofactor in one-carbon metabolism. Importantly, folate-dependent methylation processes directly impact the main mechanisms discussed earlier: DNA and histone methylation control inflammatory gene expression, antioxidant enzyme production, mitochondrial biogenesis, synaptic protein synthesis, and mTOR pathway regulation. This mechanistic framework offers a unified explanation of how genetic predisposition, environmental influences, and developmental timing merge to produce the varied clinical features seen in autism spectrum disorders [30,31,32].

Central to this model is S-adenosylmethionine (SAM), known as the “master methyl donor,” which acts as a substrate for over 100 methyltransferase reactions. Nevertheless, SAM is not just a passive substrate; cellular pools are limited and tightly regulated. This creates competitive situations in which different methyltransferases compete for SAM according to their binding affinities, cellular priorities, and functional significance [33]. Such competition becomes especially important during folate deficiency or metabolic dysfunction, when SAM levels are reduced, and cellular prioritization determines which methylation processes are sustained or compromised—impacting neuroinflammation, OS, mitochondrial function, synaptic plasticity, and mTOR signaling.

### 2.1. One-Carbon Metabolism: Biochemical Foundations

Dietary folate is mainly absorbed in the proximal small intestine—specifically the duodenum and proximal jejunum—through the proton-coupled folate transporter (PCFT/SLC46A1), which functions best in the acidic environment of the brush border. A secondary, pH-dependent pathway involves the reduced folate carrier 1 (RFC1/SLC19A1) [34,35]. Dietary polyglutamates are deconjugated by intestinal folate conjugase (glutamate carboxypeptidase II) before absorption. One-carbon metabolism (OCM) comprises two linked pathways that convert Hcy back into methionine: the remethylation pathways and the transsulfuration pathway, which irreversibly removes excess Hcy. The folate-dependent remethylation pathway, driven by methionine synthase (MTR) using 5-methyltetrahydrofolate and vitamin B12 (cobalamin), occurs in nearly all tissues but is especially crucial in the brain. The alternative, folate-independent pathway involves betaine–homocysteine methyltransferase (BHMT), which uses betaine—sourced from diet or liver choline oxidation—as a methyl donor. BHMT is predominantly expressed in the liver and kidneys. In the liver, the choline-betaine pathway is the major source of methyl groups for remethylating Hcy and regenerating SAM under normal dietary conditions. In the brain, however, the folate cycle is the primary and often the sole route for remethylation because BHMT expression is minimal. This tissue-specific division explains why brain methylation capacity is particularly vulnerable to folate deficiency, offering a biochemical explanation for the brain-specific effects discussed throughout this review.

Once formed, SAM donates its methyl group in transmethylation reactions, [36] generating S-adenosylhomocysteine (SAH), a potent product inhibitor of nearly all SAM-dependent methyltransferases. SAH is hydrolyzed by SAH hydrolase (AHCY) in a reversible reaction that favors SAH synthesis under physiological conditions and depends on efficient removal of both products, Hcy and adenosine, to proceed in the hydrolytic (SAH-consuming) direction [37,38,39]. (When Hcy remethylation (via MTR or BHMT) or transsulfuration is impaired, Hcy accumulates, driving the AHCY equilibrium back toward SAH synthesis; this is the principal biochemical mechanism by which folate or B12 insufficiency raises intracellular SAH and depresses the SAM/SAH ratio, independent of any change in SAM synthesis per se [40,41,42]. Excess Hcy that is not remethylated enters the transsulfuration pathway, where cystathionine β-synthase (CBS, vitamin B6-dependent) condenses Hcy with serine to form cystathionine, which is subsequently cleaved by cystathionase to yield cysteine, a precursor for glutathione synthesis [43]. Transsulfuration therefore links one-carbon metabolism directly to cellular antioxidant capacity, providing a redox-relevant connection between OCM dysfunction and the oxidative stress phenotypes described elsewhere in this review.

A further mechanistic feature central to the hierarchical epigenetic hypothesis is the redox chemistry of cobalamin (vitamin B12) itself. Methionine synthase relies on a cob(I)alamin intermediate that is exceptionally electron-rich and therefore highly susceptible to adventitious oxidation to catalytically inactive cob(II)alamin whenever intracellular reactive oxygen species (ROS) rise or reducing equivalents (NADPH, glutathione) are limited [44,45]. Reactivation requires a reductive methylation step, using methionine synthase reductase (MTRR) and SAM itself as the methyl donor, that regenerates methylcobalamin from cob(II)alamin [46]. Because this repair reaction consumes SAM, oxidative stress and impaired *MTHFR*-derived 5-methyltetrahydrofolate supply act synergistically to suppress methionine synthase turnover: insufficient 5-MTHF slows re-methylation of cob(I)alamin before it is oxidized, while ROS accelerates the competing oxidation reaction. This cobalt-centered redox switch couples the prevailing cellular redox environment directly to SAM output, providing a biochemical rationale, beyond *MTHFR* genotype alone, for why oxidative stress and folate–SAM metabolism are mechanistically intertwined in the proposed epigenetic hierarchy.

The folate cycle, also known as one-carbon (1C) metabolism, is a central metabolic hub that provides methyl groups essential for DNA synthesis, methylation reactions, and epigenetic regulation [28,29,47]. This intricate biochemical network operates across multiple subcellular compartments: the cytoplasm, mitochondria, and nucleus, each supporting distinct but interconnected functions critical for neurodevelopment [48]. The pathway begins with dietary folate intake in two primary forms: synthetic folic acid from fortified foods and supplements, and natural food folates (primarily 5-methyltetrahydrofolate and 10-formyltetrahydrofolate) from vegetables, legumes, and citrus fruits [49]. Recent studies show that the system’s efficiency is significantly affected by genetic polymorphisms impacting enzyme activity and substrate use at various stages [50]. The complexity extends beyond nutrient absorption, involving key junctions where the folate cycle and ASD development intersect, from intestinal absorption through cellular uptake, metabolic activation, and epigenetic regulation.

### 2.2. SAM and Methyltransferase Competition in Metabolic Dysfunction

Once folate enters the intestinal lumen, specific enteric bacteria convert it into chemical forms that can be efficiently absorbed, positioning the gut microbiome as a dual modulator of folate status. Some bacterial genera, such as Bifidobacterium and Lactobacillus, synthesize folates de novo, whereas others enzymatically convert dietary polyglutamyl folates into monoglutamyl forms suitable for uptake by enterocytes [34,51]. In ASD, a well-documented lack of microbial diversity and dysbiosis, marked by reduced levels of Bifidobacterium and Lactobacillus and increased Clostridium species, can significantly impair both folate absorption and endogenous synthesis, making this microbial imbalance a primary bottleneck limiting folate availability in children with ASD [35,51].

### 2.3. Intestinal Absorption, Microbiome, and Folate Transport Systems

After intestinal absorption, folate must be transported through the circulation and across cellular membranes, including the blood–brain barrier (BBB), via specialized transport systems encoded by genes with polymorphisms linked to ASD susceptibility. Reduced folate carrier 1 (RFC1/SLC19A1) represents the major route of folate transport into mammalian cells and is particularly critical for transfer across the choroid plexus into cerebrospinal fluid [52,53]. Located on chromosome 21q22.3, RFC1 functions as a bidirectional anion exchanger with optimal activity at physiological pH 7.4 and preferentially transports reduced folates with a Kt ≈ 3 μM [52]. The RFC1 A80G polymorphism (rs1051266) has been associated with altered ASD risk (OR = 1.41), likely through effects on folate and homocysteine levels, [54] and RFC1 expression in the intestine and brain is adaptively regulated by folate status, creating a feedback loop in which genetic variants can be amplified by nutritional deficiency [52,53].

Folate receptor alpha (FOLR1) provides a complementary, high-affinity pathway for folate uptake. FOLR1 encodes a folate-binding protein (FRα; Kd ≈ 1 nM) anchored to cell membranes via a glycosylphosphatidylinositol (GPI) linkage, mediating folate internalization through receptor-mediated endocytosis and constituting the primary mechanism for folate transport across the blood–brain barrier [55,56]. Pathogenic variants in FOLR1 cause cerebral folate transport deficiency (FOLR1-CFTD), an autosomal recessive disorder characterized by severely reduced CSF 5-MTHF levels despite normal peripheral folate [55,56]. The FOLR1 G80A polymorphism shows a strong association with ASD (OR = 1.67), and affected individuals typically present with developmental regression, intractable seizures, ataxia, hypotonia, and autism-like features beginning at 5–24 months of age [55,57]. Notably, even when systemic folate deficiency is corrected, cerebral deficiency often persists in FOLR1 variant carriers and requires high-dose folinic acid therapy that can exploit alternative RFC1-mediated transport [56,57].

FOLR1 expression is itself subject to folate-responsive promoter methylation, creating a feedback loop in which low intracellular folate can downregulate receptor availability; whether this regulatory loop is altered in ASD-associated FOLR1 methylation status remains largely unexplored and represents an open question for future biomarker studies [58,59].

Downstream of transport, dihydrofolate reductase (DHFR) catalyzes the NADPH-dependent reduction of dihydrofolate to tetrahydrofolate, the biologically active form necessary for one-carbon metabolism [60]. A common 19 bp deletion polymorphism in DHFR intron 1 (del/del genotype prevalence ≈ 23%) has been associated with altered DHFR expression, elevated unmetabolized folic acid in plasma, and paradoxically decreased red blood cell folate [61,62]. The DHFR del/del genotype shows a marginal but significant association with ASD (OR = 2.69, 95% CI: 1.00–7.28), [54] and elevated DHFR mRNA expression in del/del carriers may promote the accumulation of unmetabolized folic acid, which can impair folate transport into the brain and competitively inhibit methyltransferases [61,63]. Cognitive studies further indicate that individuals with the DHFR del/del genotype exhibit poorer memory and executive function scores, particularly under conditions of high folic acid intake, highlighting gene–nutrient interactions that may be especially relevant during neurodevelopment [61,62].

### 2.4. Folate–Methionine Cycle and SAM Synthesis

Once inside cells, folate enters the interconnected folate–methionine cycle, a metabolic network that ultimately generates SAM, the universal methyl donor. This pathway involves multiple enzymes with known polymorphisms associated with ASD that alter their activity, creating a cascade of metabolic vulnerabilities [29,51]. The enzyme *MTHFR* catalyzes the conversion of 5,10-methylenetetrahydrofolate into 5-methyltetrahydrofolate, a crucial step linking folate metabolism with the remethylation of homocysteine. 5-MTHF then transfers its methyl group to vitamin B12 to form methylcobalamin, which donates this methyl group to homocysteine, converting it back into methionine. Methionine is subsequently adenosylated by methionine adenosyltransferase (MAT) to produce SAM, and, after participating in transmethylation reactions, SAM is converted into SAH, which is hydrolyzed to regenerate homocysteine, thereby completing the cycle [28,64].

This metabolic network is highly sensitive to both genetic variation and nutritional deficits. Any impairment in these steps hampers folate-dependent pathways, leading to reduced purine and thymidylate synthesis, increased levels of homocysteine and SAH, and, critically, decreased SAM availability [28,29]. These disruptions directly affect DNA synthesis, genomic stability, and, most importantly in the context of ASD, cellular methylation capacity, thereby altering epigenetic processes that regulate neurodevelopment.

## 3. Epigenetic Consequences of Folate Metabolism Dysfunction

The dependence of cellular methylation reactions on SAM synthesis and availability enables cells to sense metabolic and nutritional state and respond by altering genome-wide expression patterns through epigenetic modifications [28,65]. This tight connection between folate metabolism and epigenetic regulation operates through several interconnected mechanisms that influence ASD risk and neurodevelopmental trajectories.

Folate deficiency affects DNA methylation regulation by decreasing SAM levels, leading to extensive DNA hypomethylation. This includes both CpG-island hypomethylation and, in post-mitotic neurons, alterations in non-CpG (CpH) methylation, which is unusually abundant in neuronal genomes and contributes substantially to neuronal gene regulation; both marks are considered relevant, non-interchangeable readouts of OCM-driven epigenetic dysregulation in this review. In ASD, abnormal DNA methylation patterns are well recognized, with folate-associated genetic variants such as *MTHFR* C677T and MTRR A66G showing strong connections to hypomethylation phenotypes. Individuals with the *MTHFR* 677TT genotype exhibit the lowest methylation levels, higher homocysteine concentrations, and lower plasma folate levels. This hypomethylation can hinder the regulation of vital neurodevelopmental genes, interfere with imprinting, and alter the expression of synaptic proteins, neurotransmitter receptors, and transcription factors essential for cortical development [29,54,65,66,67].

Histone methylation dynamics extend beyond DNA methylation, with folate metabolism significantly impacting histone methylation patterns that regulate chromatin accessibility and gene expression. Evidence shows that folate deficiency reduces specific histone methylation marks crucial for neurodevelopment, including H3K79me2, H3K4me3, and H3K27me3. Research on neural tube defects (NTDs), a distinct folate-dependent neurodevelopmental disorder, indicates that folate deficiency reduces DOT1L-mediated H3K79me2 methylation at promoters of key genes such as SHH and SUFU, leading to abnormal neural patterning [68]. H3K79me2 levels are directly linked to maternal serum folate and DOT1L expression, with folinic acid supplementation restoring normal H3K79me2 levels in this NTD model; this evidence derives from an NTD model rather than direct ASD data and should not be extrapolated to ASD without independent confirmation. Similarly, folate deficiency decreases H3K4me1 and alters H3K27 dimethylation at neurodevelopmental gene sites, while folate supplementation helps normalize these repressive epigenetic marks [68].

These histone methylation effects are primarily driven by SAM availability: histone methyltransferases (HMTs) rely on SAM as the methyl donor for methylating histone lysines and arginines. Under folate deficiency, decreased SAM synthesis leads to competition between DNA methyltransferases (DNMTs) and HMTs, with specific patterns of vulnerability influenced by enzyme kinetics, cellular levels, and developmental stage (as explained in the SAM hierarchy Section 5) [47,69]. Importantly, the HMTs most affected by SAM scarcity—such as G9a/EHMT2 (H3K9me2), EZH2 (H3K27me3), and SETD1A (H3K4me3)—are also those linked to ASD risk through genome-wide association and functional research.

During critical neurodevelopmental stages, the epigenetic impact of folate deficiency becomes especially significant, as DNA and histone methylation patterns are being formed [70,71]. Studies of nearly 1000 human brains indicate that DNA methylation varies most significantly before birth, emphasizing the activation of pathways essential for cortical development. Genes linked to autism show particularly notable DNA methylation shifts during prenatal brain growth, highlighting their vital roles in cortical formation and suggesting that disruptions, like those from folate deficiency, could contribute to ASD development [72].

Maternal folate levels around conception have been shown to affect the offspring’s epigenome by influencing DNA methylation at imprinted loci and retrotransposons, leading to long-term effects on gene expression and traits into adulthood [73,74]. Epidemiological studies support this developmental programming idea: maternal intake of folic acid during pregnancy (≥600 μg daily) is linked to a significant decrease in ASD risk (OR = 0.64–0.73), especially when supplementation happens during the periconceptional window (one month before to one month after conception) [59,75]. Importantly, this protective effect varies with the maternal *MTHFR* genotype, with greater benefits observed in mothers carrying the 677T variant—those with lower *MTHFR* activity and greater sensitivity to folate levels [59,75].

The relationship between genetic variations in folate metabolism, nutritional folate levels, and epigenetic regulation generates complex gene–nutrient interactions that influence ASD risk [76,77]. For example, the *MTHFR* C677T polymorphism is associated with ASD primarily in countries without mandatory folic acid fortification, but not in countries with such programs [62,75]. This gene-environment interaction indicates that adequate folate intake can offset genetic vulnerabilities in the folate cycle, likely by ensuring sufficient SAM production to support DNA and histone methylation, even with reduced *MTHFR* activity.

Similarly, the RFC1 G allele is more common among mothers of children with ASD (but not in affected children or fathers), indicating that maternal folate transport during pregnancy plays a critical role in neurodevelopmental outcomes via epigenetic programming mechanisms [59,78]. The DHFR 19 bp deletion polymorphism also interacts with nutrients, with del/del carriers experiencing cognitive impairments mainly when consuming high levels of folic acid. This is likely due to the buildup of unmetabolized folic acid, which can disrupt normal folate balance and epigenetic regulation [61,62].

**Figure 2:**
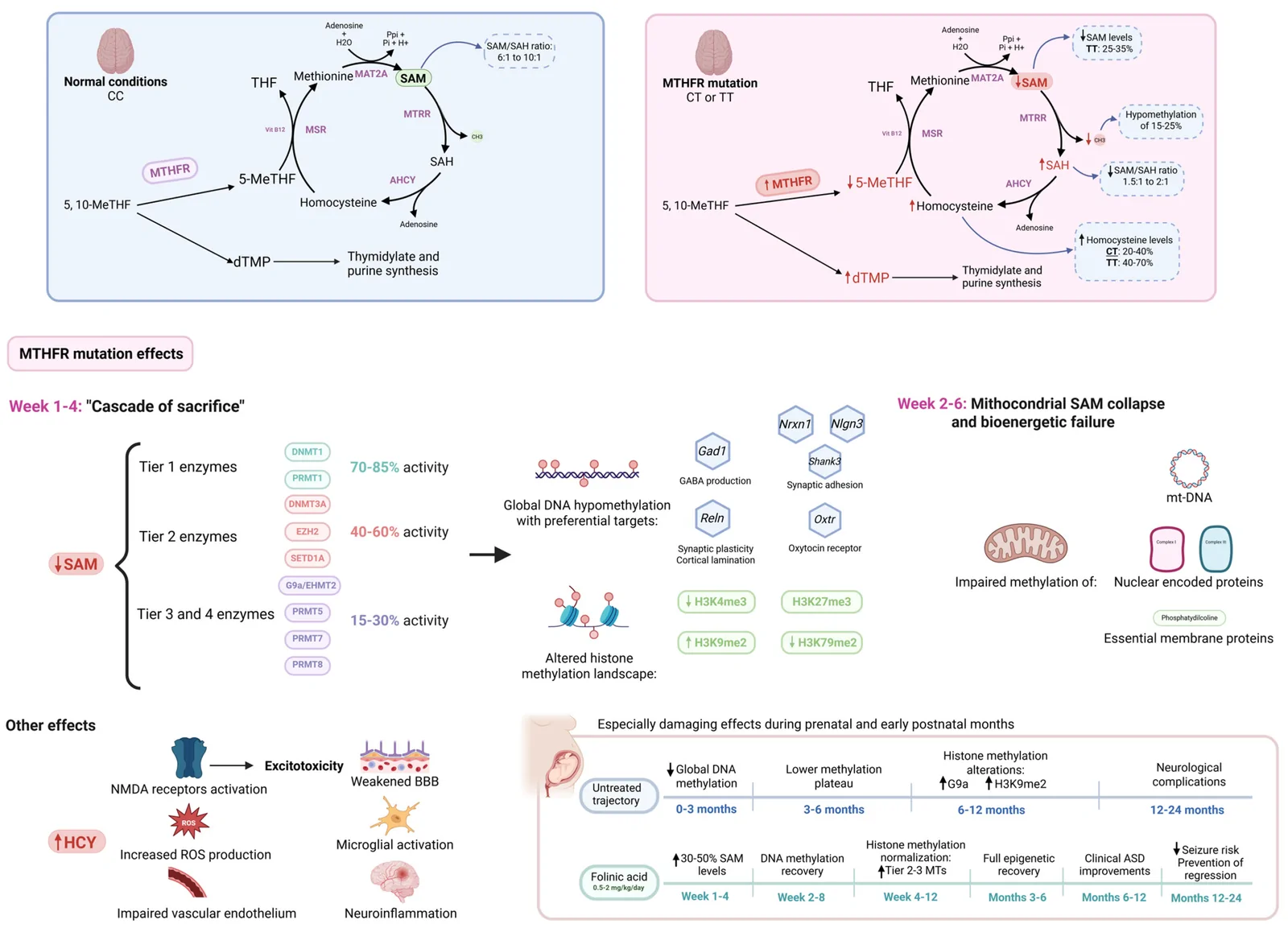
Impact of *MTHFR* mutation on the methionine cycle and downstream molecular pathways, including SAM-dependent methyltransferase activity, homocysteine imbalance, and mitochondrial dysfunction. Administration of folinic acid restores methylation-related pathways and ameliorates ASD-related molecular alterations.

This evidence supports a model in which folate metabolism links genetic predispositions, dietary intake, and epigenetic regulation, thereby influencing neurodevelopmental outcomes. Variations in genes involved in folate transport, activation, or utilization can create metabolic bottlenecks, lowering SAM levels during key developmental periods. This reduction may cause abnormal DNA and histone methylation, impacting genes important for synaptic growth, neuronal connectivity, and social behavior. Recognizing these gene-nutrient-epigenetic relationships provides a scientific basis for personalized folate supplementation strategies informed by genetic test results (Figure 2).

### 3.1. MTHFR Polymorphisms and Population-Specific Effects: The Primary Genetic Gateway to Epigenetic Dysregulation in ASD

Methylenetetrahydrofolate reductase (*MTHFR*) is a key metabolic and epigenetic enzyme that links folate intake to SAM production [50,79]. This, in turn, affects the epigenetic system’s ability to sustain normal chromatin states during neurodevelopment. Variations in the *MTHFR* gene serve as the main link between genetic susceptibility and epigenetic issues in ASD [62,65,80,81,82].

*MTHFR* is the key enzyme controlling folate metabolism. It catalyzes the irreversible, FAD-dependent reduction of 5,10-methylenetetrahydrofolate to 5-methyltetrahydrofolate (5-MTHF), which is critical for the remethylation of homocysteine to methionine [28,83]. This step marks a vital metabolic decision point: 5,10-methylenetetrahydrofolate can either be converted to 5-MTHF for homocysteine remethylation and SAM production or used for thymidylate and purine synthesis needed for DNA replication [28]. Therefore, *MTHFR* activity influences the balance between nucleotide synthesis and methylation capacity, affecting genomic stability and epigenetic regulation. When *MTHFR* activity is reduced, 5-MTHF levels decrease, impairing homocysteine remethylation and limiting SAM production. At the same time, more 5,10-methylenetetrahydrofolate is diverted to nucleotide synthesis, creating a trade-off between DNA replication and epigenetic methylation [28].

Several studies have reported associations between genetic polymorphisms in the folate pathway and homocysteine (Hcy) metabolism, with *MTHFR* representing the most extensively studied gene. Different SNPs have been described in the *MTHFR* gene, with C677T (rs1801133, Ala222Val) and A1298C (rs1801131, Glu429Ala) being the two most frequent polymorphisms shown to reduce enzyme activity [81,82].

### 3.2. C677T Polymorphism: Molecular Mechanisms and Functional Consequences

The C677T polymorphism results in an alanine-to-valine substitution at position 222 in the catalytic domain of *MTHFR*, producing a thermolabile enzyme variant with dramatically reduced activity: 30% reduction in heterozygotes (CT) and 60% reduction in homozygotes (TT) compared to wild-type (CC) [81,82,83]. This thermolability manifests as progressive loss of FAD cofactor binding and accelerated enzyme degradation at physiological temperatures, with enzyme instability increasing significantly above 37 °C, creating vulnerability during fever or metabolic stress [81,84].

The functional consequence is impaired conversion of 5,10-methylenetetrahydrofolate to 5-MTHF, leading to several interconnected metabolic abnormalities that create a cascade of epigenetic dysregulation [42,85].

#### 3.2.1. Elevated Homocysteine (Hyperhomocysteinemia)

A deficiency in 5-MTHF reduces the efficiency of the methionine synthase (MS) reaction, which depends on methyl-5-MTHF and vitamin B12 as cofactors to convert homocysteine into methionine. Plasma homocysteine levels rise by 20–40% in CT carriers and 40–70% in TT carriers compared to the CC genotype [86]. Elevated homocysteine has several harmful effects on the developing brain: it activates NMDA receptors, leading to excitotoxicity, increases ROS production through NADPH oxidase activation, impairs vascular endothelial function and weakens blood–brain barrier integrity, and stimulates microglial activation and neuroinflammation [80,87].

#### 3.2.2. Reduced SAM Production

Impaired methionine regeneration via homocysteine remethylation decreases the substrate for MAT, which converts methionine into SAM [88]. Children with autism and the *MTHFR* 677TT genotype have plasma SAM levels approximately 25–35% lower than those of CC genotype controls [89]. This SAM depletion rapidly impacts all methylation reactions that rely on SAM, as detailed in the SAM hierarchy Section 5.

#### 3.2.3. Increased SAH Buildup and Unfavorable SAM/SAH Ratios

Decreased SAM production, combined with ongoing cellular methylation, creates an imbalance in adenosine metabolism. SAH, generated as a byproduct of methyltransferase activity, accumulates because a compromised remethylation cycle hinders its conversion back to homocysteine [28]. This results in a significantly unfavorable SAM/SAH ratio: healthy individuals typically have ratios of 6:1 to 10:1, whereas *MTHFR* 677TT individuals with autism often display ratios between 1.5:1 and 2:1 [64,90]. SAH acts as a competitive inhibitor at the SAM-binding site of SAM-dependent methyltransferases; because Ki(SAH) values differ substantially across DNMTs, HMTs, and PRMTs, the practical impact of a given SAM/SAH ratio on methylation capacity varies by enzyme, and a single universal inhibition constant should not be assumed across this structurally diverse enzyme superfamily [91]. This creates a “double hit”: reduced SAM levels and the inhibitory effects of accumulated SAH together worsen the methylation deficit.

#### 3.2.4. DNA and Histone Hypomethylation

Reduced SAM synthesis coupled with elevated SAH leads to a significant decrease in cellular methylation capacity. In cortical tissue from *MTHFR* 677TT individuals, genome-wide DNA methylation (i.e., 5-methylcytosine content) drops by 15–25%, with especially strong hypomethylation observed at CpG islands within gene promoters [65,92]. This widespread DNA hypomethylation impacts various essential gene groups: those regulating neuroinflammation (such as IL-6, TNF-α, IL-1β promoter regions), antioxidant defenses (including SOD2 and glutathione S-transferase genes), mitochondrial biogenesis (targets like TFAM, NRF1), synaptic function (NRXN1, NLGN3, SHANK3), and neurotransmitter systems (GAD1, RELN) [93].

The influence extends beyond DNA methylation to include histone methylation. The *MTHFR* 677TT genotype is associated with decreased H3K4me3 (an active mark) at neurodevelopmental gene promoters and, unexpectedly, increased H3K9me2 (a repressive mark) in specific chromatin regions, suggesting dysregulation of histone methyltransferases during SAM deficiency [47,87]. This aligns with the SAM hierarchy model: during folate deficiency, Tier 1–2 histone methyltransferases (such as SETD1A for H3K4me3 and EZH2 for H3K27me3) are partly preserved, while Tier 3 enzymes (like G9a for H3K9me2) are upregulated as a compensation despite low SAM levels, resulting in an abnormal chromatin landscape [47,94].

### 3.3. A1298C Polymorphism and Compound Heterozygosity

The A1298C polymorphism (Glu429Ala, rs1801131), which is in the regulatory domain of *MTHFR*, results in a moderate reduction in enzyme activity—around 35% in heterozygotes and 60% in homozygotes—when compared to C677T [95]. Although A1298C alone has a weaker association with ASD (OR = 1.25–1.35), having both mutations (C677T/A1298C genotype) can produce additive or synergistic effects, further impairing *MTHFR* enzyme function and disrupting metabolic processes [95,96].

Individuals with the compound heterozygous C677T/A1298C genotype (about 5–8% of the population) exhibit a 65–75% reduction in *MTHFR* activity, similar in severity to that of the homozygous 677TT genotype [86]. The C677T/A1298C genotype demonstrates that homocysteine levels in these individuals fall intermediate between those observed in the CT/AA and TT genotypes, with elevations of 30–50% above the CC reference range. SAM/SAH ratios are comparable to those found in homozygous 677TT carriers, indicating similarly disrupted methylation potential [97]. Furthermore, global DNA methylation deficits in compound heterozygotes approach those seen in homozygous TT individuals, suggesting substantial impairment in epigenetic regulation. Additionally, individuals with the C677T/A1298C genotype demonstrate enhanced sensitivity to folate nutrient deficiency, making them particularly vulnerable to insufficiency in this critical cofactor.

Recent therapeutic research has shown that treatment responses vary by *MTHFR* genotype. A significant 2025 study involving 76 Chinese children with ASD (average age 4.2 years) explored how the efficacy of folinic acid varies among different *MTHFR* genotypes [98]. Children carrying the *MTHFR* A1298C mutation, specifically those with the AC/CC genotype, demonstrated markedly superior improvements across multiple developmental domains following high-dose folinic acid treatment (1.0–2.0 mg/kg/day) compared to wild-type carriers [98]. In visual–motor imitation, 78% of A1298C carriers exhibited meaningful improvement compared to only 52% of wild-type individuals (*p* = 0.018). Social reciprocity improved in 71% of mutation carriers compared with 38% of the CC genotype group (*p* = 0.025). Communication skills improved in 82% of A1298C carriers, compared with 45% in wild-type individuals (*p* = 0.009). Gross and fine motor skills demonstrated improvement in 69% of carriers compared to 41% in the CC genotype group (*p* = 0.031). Most notably, the adaptive behavior composite score increased by an average of 18.3 ± 7.2 points in A1298C carriers compared to 8.1 ± 5.4 points in control subjects (*p* = 0.003).

Children with the MTRR A66G mutation (AG/GG genotype) showed greater improvements in cognitive, verbal, and preverbal skills after treatment, with 73% experiencing clinically meaningful language gains (an increase of at least 1.5 standard deviations) [98]. In contrast, only 31% of those with the AG/AA genotype showed similar progress. These findings suggest that individuals with A1298C or MTRR variants may have a greater capacity to benefit from folate-based therapies, likely because their enzymatic activity can still respond effectively to high-dose supplementation that saturates the substrate, despite potential impairments in enzyme kinetics. The study also revealed that different combinations of alleles can unexpectedly yield stronger responses to high-dose folinic acid, suggesting a compensatory mechanism in which decreased enzyme activity increases sensitivity to substrate supplementation.

#### C677T Polymorphism: Population Genetics and Meta-Analytic Evidence

Beyond these molecular mechanisms, the clinical significance of the C677T polymorphism becomes clear when reviewing large population studies that assess its effect on ASD risk across different genetic backgrounds and nutritional environments. A systematic review and meta-analysis of 40 studies involving healthy women of reproductive age found a consistent gene-dose association between the *MTHFR* 677C > T polymorphism and blood folate concentrations, following the pattern CC > CT > TT. In studies using the microbiological assay, serum/plasma folate was 13% lower in individuals with the TT genotype than in those with the CC genotype (95% credible interval: 7–18%), whereas red blood cell folate was 16% lower (95% credible interval: 12–20%). TT homozygosity is also associated with higher plasma homocysteine, particularly when folate status is low. Separately, an observational study found lower global DNA methylation in individuals with the 677TT genotype, but this difference was restricted to participants with low folate status, indicating a gene–nutrient interaction rather than an invariant effect of genotype. These alterations may be biologically relevant during development, although direct evidence linking genotype-associated global hypomethylation to neurodevelopmental outcomes remains limited [99,100].

Recent large-scale studies have provided unprecedented insights into how the *MTHFR* C677T variant affects ASD risk across populations. A meta-analysis comprising 2258 ASD cases and 2073 controls from six studies investigated the association between the *MTHFR* C677T polymorphism and ASD susceptibility in Chinese Han populations [101]. Significant associations were observed across all five genetic models: allelic (T vs. C: OR = 1.89, 95% CI: 1.27–2.79; *p* = 0.001), homozygous (TT vs. CC: OR = 2.44, 95% CI: 1.43–4.15; *p* < 0.001), heterozygous (CT vs. CC: OR = 1.73, 95% CI: 1.19–2.51; *p* = 0.004), dominant (CT + TT vs. CC: OR = 2.03, 95% CI: 1.31–3.15; *p* = 0.002), and recessive (TT vs. CT + CC: OR = 1.95, 95% CI: 1.21–3.13; *p* = 0.006). These estimates correspond to 89% higher odds for the T allele, 144% higher odds for the TT genotype relative to CC, 73% higher odds for CT relative to CC, 103% higher odds under the dominant model, and 95% higher odds under the recessive model. Subgroup analyses indicated that the associations were significant in Northern Han populations but not in Southern Han populations [101].

Notably, this association was particularly strong in Northern Han Chinese populations (OR = 2.89 for TT vs. CC) but not statistically significant in Southern Han populations (OR = 1.42, 95% CI 0.92–2.20, *p* = 0.11), demonstrating striking population-specific effects [102]. The minor allele frequency (T allele) differed significantly between regions: 45% in Northern Han vs. 39% in Southern Han, indicating population stratification. This geographic variation likely reflects gene-environment interactions: Northern China has demonstrably lower dietary folate intake (mean intake 174 ± 45 μg/day) and lower vitamin B12 consumption compared to Southern regions with traditional diets richer in vegetables and legumes (mean intake 287 ± 62 μg/day), creating an environmental context where the C677T variant exerts greater phenotypic impact [103]. Additionally, Northern regions have historically had lower rates of prenatal folic acid supplementation (approximately 22% in 2000, increasing to 68% by 2015), while Southern regions adopted supplementation programs earlier and more extensively [103].

Global meta-analyses confirm these findings across diverse populations. A comprehensive study of 1672 ASD patients and 6760 controls demonstrated that C677T polymorphism was associated with significantly increased ASD risk (OR = 1.50–1.89 across different genetic models) [62]. Critically, stratified analysis revealed that this association was significant only in children from countries without mandatory food fortification with folic acid (OR = 1.97, 95% CI 1.45–2.68), but not in countries with fortification programs (OR = 1.12, 95% CI 0.79–1.58) [62,75]. This gene-nutrient interaction provides compelling evidence that adequate folate nutrition can compensate for genetic vulnerabilities in *MTHFR* activity, likely by providing sufficient substrate (5,10-methylenetetrahydrofolate) to maintain 5-MTHF production despite reduced enzyme efficiency. The mechanism involves substrate saturation: even a thermolabile enzyme with reduced Vmax retains most of its catalytic capacity at high substrate concentrations; therefore, adequate dietary folate ensures sufficient 5-MTHF production to support methylation demands [104].

This finding has profound public health implications: it demonstrates that population-level dietary fortification can effectively eliminate the excess ASD risk associated with *MTHFR* C677T genotype, suggesting that genetic vulnerability is not deterministic but rather conditional on nutritional context. Countries that implemented mandatory folic acid fortification programs (United States, Canada, Australia, UK, South Korea) between 1995 and 2005 have shown the greatest reduction in ASD-folate associations, while countries without mandatory fortification show more heterogeneous C677T-ASD associations. This heterogeneity likely reflects differing baseline dietary folate intake rather than policy neglect: several Mediterranean and other non-fortifying countries (e.g., Spain, Italy, France) have historically adequate dietary folate intake from vegetables and legumes and have not been shown to have the same degree of population folate insufficiency as pre-fortification North America. The geographic pattern in C677T-ASD associations should therefore be interpreted in light of actual population folate status and dietary patterns rather than fortification policy alone [75,105].

## 4. Epigenetic Mechanisms Connecting MTHFR Variants to ASD: The Cascade Model

The molecular pathway linking reduced-function *MTHFR* variants to ASD susceptibility may involve epigenetic dysregulation secondary to altered methyl-donor availability. Reduced *MTHFR* activity can limit 5-methyltetrahydrofolate production, potentially impairing methionine regeneration and SAM-dependent methylation, particularly under conditions of low folate or vitamin B12 availability. Consistently, individuals with ASD show lower peripheral SAM levels and SAM/SAH ratios, although these abnormalities have not been specifically attributed to *MTHFR* genotype. ASD studies have also identified tissue- and locus-specific alterations in DNA and histone regulation involving neurodevelopmental genes, rather than uniform global hypomethylation. Reduced mitochondrial SAM availability may additionally impair complex I stability, iron–sulfur-cluster synthesis, and oxidative phosphorylation, but a direct causal link between common *MTHFR* variants, mitochondrial dysfunction, and ASD remains unproven. These mechanisms may be particularly relevant during prenatal and early postnatal development, consistent with observational associations between periconceptional maternal folate supplementation and reduced ASD risk in offspring [106,107,108,109,110].

### 4.1. Longitudinal Changes in Methylation During MTHFR-Related Folate Deficiency

Prospective studies of folate-supplemented versus unsupplemented children carrying *MTHFR* C677T variants report divergent trajectories: supplementation is associated with earlier normalization of SAM/SAH ratios and global DNA methylation and with better developmental outcomes, whereas untreated cohorts show more persistent methylation deficits and higher rates of neurological complications, including seizures and developmental regression [93,111].

### 4.2. Gene-Nutrient-Epigenetic Interactions: The Compensation Model

The *MTHFR* C677T polymorphism exemplifies a broader principle of gene-nutrient-epigenetic interaction: genetic variants that impair enzymatic function can be compensated for by environmental nutritional intervention if substrate is provided in sufficient quantities. This compensation operates through several mechanisms:

In the substrate saturation mechanism, even a thermolabile *MTHFR* enzyme with reduced maximum velocity (Vmax) retains meaningful catalytic capacity at high substrate concentrations. When dietary folate is adequate (≥600 μg daily), the 5,10-methylenetetrahydrofolate concentration remains sufficient to drive the *MTHFR* reaction near maximal velocity despite the enzyme’s compromised kinetics [104,112]. Conversely, when dietary folate is marginal (200–400 μg daily), substrate limitation becomes rate-limiting, and the enzyme’s reduced Vmax becomes apparent [112].

When *MTHFR* activity is impaired, alternative routes to 5-MTHF become upregulated as a secondary compensatory response. Folate monoglutamate can directly accept a methyl group from betaine (via betaine–homocysteine methyltransferase, BHMT) to regenerate methionine without requiring the *MTHFR* reaction [113]. However, BHMT activity is limited by betaine availability and is primarily active in the liver, not in neural tissue, making it an incomplete compensatory mechanism [113].

The most clinically relevant compensation mechanism involves folinic acid (leucovorin, 5-formyl-tetrahydrofolate (5-Formyl-THF)) supplementation. Folinic acid (5-formyl-THF) is first converted to 5,10-methenyl-THF by 5-formyltetrahydrofolate cyclo-ligase (MTHFS), replenishing the intracellular THF-polyglutamate pool; it can also generate 5,10-methylene-THF and other one-carbon-substituted folates that support purine and thymidylate synthesis without requiring further *MTHFR*-catalyzed reduction. Importantly, conversion to 5-MTHF specifically still requires *MTHFR* activity; the clinical benefit of high-dose folinic acid supplementation (0.5–2.0 mg/kg/day) in *MTHFR*-deficient individuals is therefore better explained by substrate saturation of residual (thermolabile) *MTHFR* activity and by folate-pool replenishment supporting *MTHFR*-independent one-carbon reactions rather than a complete bypass of the *MTHFR* step [114]. This explains why *MTHFR* 677TT individuals respond dramatically to folinic acid treatment despite having permanently impaired *MTHFR* activity [93].

At the population level, mandatory folic acid fortification programs create a gene-environment interaction that effectively eliminates the *MTHFR*-ASD association. By increasing average dietary folate intake from ~190 μg/day (pre-fortification) to ~600+ μg/day (post-fortification), fortification ensures that even individuals with low *MTHFR* enzyme activity maintain adequate 5-MTHF production [105,115]. This explains the geographic variation observed in *MTHFR*-ASD studies: in countries without mandatory fortification, *MTHFR* C677T shows a strong association with ASD (OR = 1.97–2.44), while in countries with fortification programs, the association is eliminated (OR = 1.12) [62,105].

### 4.3. Clinical and Neurobiological Implications of Epigenetic Dysregulation Caused by MTHFR

Epigenetic modifications associated with *MTHFR* genetic variants lead to distinct neurobiological and behavioral traits in individuals with autism [103,116]. Social cognition deficits are linked to hypomethylation and dysregulation of RELN and OXTR promoters, along with changes in H3K4me3 at these gene locations, resulting in impaired cortical lamination through RELN dysfunction, reduced oxytocin receptor expression from OXTR hypomethylation, decreased social information processing in the prefrontal cortex, and deficits in theory of mind and social reciprocity. Notably, there is a significant correlation between promoter methylation status and social reciprocity scores (r = 0.67), demonstrating that the degree of aberrant methylation at these critical gene loci predicts the magnitude of social–cognitive impairment [116,117].

Communication and Language Delays: Temporal cortex dysregulation of CNTNAP2 and NRXN1 (synaptic connectivity genes) via hypomethylation and altered histone marks, combined with dysregulated expression of genes involved in neurotransmitter systems (e.g., hypomethylation of GAD1 driving GABAergic imbalance) [93,103]. Concurrent hypomethylation of GAD1, which encodes glutamate decarboxylase involved in GABAergic neurotransmitter synthesis, drives GABAergic imbalance that disrupts the excitatory–inhibitory balance necessary for proper neural communication. These epigenetic dysregulations manifest as delayed speech and language development, atypical prosody with pragmatic language impairments, and difficulty in processing auditory information properly. Notably, a significant correlation exists between the severity of language delay and methylation patterns in the temporal cortex (r = 0.61 between temporal cortex methylation and language scores), demonstrating that the degree of epigenetic dysregulation in language-critical brain regions directly predicts the magnitude of language-developmental impairment [87,93].

Seizure susceptibility in individuals with *MTHFR* dysfunction arises from convergent epigenetic and metabolic dysregulation that affect multiple neural systems. GABAergic dysregulation driven by GAD1 hypomethylation, combined with resulting excitatory–inhibitory imbalance, creates heightened neuronal excitability. This is compounded by mitochondrial dysfunction characterized by impaired oxidative phosphorylation, which reduces neuronal energy availability and further compromises the capacity to maintain inhibitory tone. Collectively, these mechanisms increase vulnerability to seizure threshold breaching. Clinically, approximately 15–20% of untreated children carrying the *MTHFR* 677TT genotype develop seizures during early childhood. Notably, seizure risk is dramatically reduced by approximately 70% with folinic acid supplementation, demonstrating that correcting one-carbon metabolism dysfunction substantially mitigates seizure vulnerability. Seizures typically emerge between 8 and 18 months of age, a critical developmental window characterized by active synaptic pruning and cortical maturation, suggesting that the timing of metabolic dysfunction during peak neurodevelopmental reorganization represents a key determinant of seizure susceptibility [93,118].

Developmental regression triggered by progressive mitochondrial SAM depletion and secondary mTOR pathway dysregulation is characterized by loss of previously acquired social and communication skills, behavioral deterioration, and increased repetitive behaviors. This condition occurs in 8–12% of untreated *MTHFR* 677TT children and is highly responsive to folinic acid intervention, with skill recovery observed in 75–80% of cases [93,118].

Intellectual disability features arising from global epigenetic dysregulation, particularly affecting genes important for dendritic spine maturation and synaptogenesis, manifest as mild to moderate intellectual disability with an IQ range between 40 and 70, characterized by disproportionate impairment of verbal cognition while motor and visuospatial abilities remain relatively preserved. Notably, intellectual disability in this context demonstrates responsiveness to folinic acid intervention, with cognitive gains of 8–15 IQ points typically observed over a 12-month treatment period [93].

### 4.4. MTHFR as a Mechanistic Gateway to ASD

The *MTHFR* C677T polymorphism illustrates how a single genetic change can lead to epigenetic misregulation by limiting SAM production, resulting in autism-related neurobiological issues. However, this pathway is not predetermined: environmental factors like sufficient maternal folate and postnatal folinic acid supplementation can offset genetic risks if administered during key developmental periods. This understanding shifts the view of *MTHFR*-linked ASD from an unavoidable genetic fate to a condition that may in principle be amenable to prevention or treatment through personalized medicine, combining genetic screening, developmental neurobiology, and specific nutritional interventions.

The notable discovery that *MTHFR*-ASD links disappear in populations with mandatory folic acid fortification shows how population-wide nutritional interventions can successfully remove genetic risks. This provides strong evidence for precision public health strategies targeting complex neurodevelopmental disorders (Table 1).

#### Synergistic Genetic Interactions

Recent evidence reveals that *MTHFR* polymorphisms do not act in isolation but interact synergistically with other folate pathway variants. MTRR is an enzyme that catalyzes, in a vitamin B12-dependent manner, the remethylation of homocysteine to methionine. The combination of *MTHFR* C677T and MTRR A66G polymorphisms exhibits significant effects on homocysteine accumulation and methylation capacity. Patients carrying both variants demonstrate the most severe metabolic dysfunction [119] and a higher risk of suffering ASD due to the accumulation of homocysteine [57].

The same tiered vulnerability logic applies at the level of gene–gene interaction: variants that further lower SAM availability (e.g., combined *MTHFR*/MTRR polymorphisms) are expected to preferentially compromise Tier 3–4 methyltransferases (including G9a/EHMT2), while Tier 1 housekeeping methylation is comparatively protected, offering a candidate mechanistic explanation for the selective, rather than global, pattern of epigenetic disruption reported in ASD; see the Section 5.1 below for the full evidentiary basis.

This hierarchy is proposed as one possible explanation for why folate-related methylation disturbances could preferentially affect social and language phenotypes in some ASD subgroups: the epigenetic prioritization system selectively protects motor circuits while preferentially disrupting social/language circuits. This is not because motor neurons are inherently more resilient but because the genes regulating motor function are constitutively maintained by Tier 1 mechanisms, while genes regulating social behavior are dynamically regulated by Tier 3 mechanisms that are vulnerable to SAM depletion.

## 5. From Folate to Methylation: The SAM-Dependent Hierarchy

SAM is, among other things, the main biological source of methyl groups, which play a vital role in cellular growth, differentiation, and function. It is considered to be surpassed only by ATP in the number of reactions it serves as a cofactor. Its synthesis takes place in the liver during the methionine cycle, which is functionally linked to the folate cycle.

Under physiological conditions, most of the SAM generated is used in transmethylation reactions, where it is converted to SAH. SAH is a potent competitive inhibitor of these reactions; therefore, its conversion to homocysteine is critical. This homocysteine can follow different pathways, including one catalyzed by methionine synthase using folate and vitamin B12, and another one catalyzed by betaine–homocysteine methyltransferase [64].

The ratios of SAM to SAH and their absolute levels are commonly used indicators of methylation capacity. In Table 2, the summarized values should be interpreted as literature-based subgroup profiles that require explicit source mapping and, where source-specific confirmation is unavailable, as approximate integrative summaries rather than definitive pooled estimates.

These results show that children with ASD have a 54.3% lower SAM/SAH ratio compared to typically developing controls (2.73 versus 5.97). The greatest metabolic disruption is observed in children with additional conditions like language delay, seizures, and autoimmune issues (FRAA+) [109]. Folate receptor autoantibodies (FRAAs) appear to exacerbate this imbalance, significantly reducing methylation capacity.

### 5.1. Molecular Mechanisms of Competitive SAM Utilization

The distribution of SAM among competing methyltransferases forms a complex cellular prioritization system. This system has evolved to safeguard vital functions during metabolic stress. It operates through multiple overlapping mechanisms, not just simple kinetic competition. These include intricate regulatory networks, subcellular compartmentalization, and adaptable prioritization systems that adjust based on developmental stage and stress conditions.

The prioritization hierarchy of methyltransferases, summarized in Table 3, should be interpreted as a working model derived from biochemical properties, relative abundance, subcellular context, and indirect neurodevelopmental evidence rather than as a directly validated hierarchy in human ASD tissue: Tier assignment combines Km(SAM) with factors like cellular abundance, subcellular localization, and expression stability during metabolic stress. As a result, two enzymes with similar Km ranges may be assigned to different tiers if their abundance or compartmentalization differs significantly. This classification relies on multiple parameters, not just Km, which is a deliberate modeling limitation acknowledged in this review.

#### The Three-Tier Priority System

Unless specified otherwise, the activity percentages listed refer to the estimated catalytic activity of individual enzyme molecules when SAM availability is reduced. They do not represent the total cellular methyltransferase flux or genome-wide mark deposition, both of which are also affected by enzyme abundance and potential compensatory mechanisms. The cited percentage activity losses are derived from in vitro enzyme kinetic data (Km, Vmax) and heterologous expression systems rather than direct measurements in ASD-relevant neuronal tissue under physiological SAM depletion. Validating these findings directly in patient-derived neurons or ASD animal models remains an important gap.

Tier 1 Enzymes (Constitutively Protected): This group includes DNMT1, PRMT1, and tRNA methyltransferases. They sustain low Km values (0.5–3.0 μM) and are safeguarded by multiple mechanisms such as high cellular abundance, sequestration within subcellular compartments, and formation of regulatory complexes. Even during SAM depletion, these enzymes retain 70–85% of their baseline activity, ensuring that critical cellular processes like DNA maintenance and protein synthesis continue effectively.

Tier 2 Enzymes (Developmentally Sensitive): DNMT3A, EZH2, and SETD1A are methyltransferases important for development but regulated by context. Their activity varies with developmental stage and cellular environment. They are prioritized during key periods for synaptic development but decline by 40–60% under metabolic stress. Their high folate sensitivity, indicated by high Km or folate dependency, makes them especially vulnerable during folate deficiency.

Tier 3 and 4 enzymes—G9a/EHMT2, PRMT5, PRMT7, and PRMT8—are key vulnerability points in the methylation system. These enzymes typically increase with stress and activity but become inadequately protected when SAM levels drop. For example, G9a/EHMT2, whose protein expression is paradoxically upregulated in ASD models and which is crucial for synaptic plasticity, is estimated to show a 50–70% reduction in per-molecule catalytic activity under SAM-limited conditions, based on Michaelis–Menten kinetics (given its comparatively high Km(SAM)). Because increased enzyme abundance can partially offset reduced per-molecule turnover, the net effect on total cellular H3K9me2 deposition is less certain and should be considered a testable prediction rather than an established quantitative outcome. Likewise, PRMT7 and PRMT8, essential for preventing intellectual disability and regulating social behavior, lose nearly all activity under metabolic stress, remaining at just 30–40%. Km(SAM) values and tier classifications for these enzymes are obtained from the enzyme kinetics literature listed in Table 3.

### 5.2. Subcellular Compartmentalization of SAM and Its Relevance to ASD

SAM distribution across cellular compartments creates distinct metabolic environments, each of which may influence neurodevelopment. Table 4 combines established biochemical knowledge on compartmentalization with inferential links to ASD and should therefore be read as a mixed-evidence table rather than a direct ASD-specific measurement set.

Nuclear compartments contain the highest levels of SAM (100–200 μM), mainly in euchromatin and nucleolar areas, underscoring their crucial role in transcription and translation during neural growth. Conversely, the mitochondrial compartment, with only 20–50 μM SAM, is more vulnerable during folate shortages or metabolic disturbances. During ASD-related folate deficiency, SAM levels decrease more significantly in mitochondrial and cytoplasmic compartments, while nuclear stores are maintained by MAT2A-mediated import. This failure of compartmentalization seems especially relevant to ASD, resulting in a paradox: euchromatic gene activation (Tier 1–2 functions) may be somewhat preserved, whereas mitochondrial metabolism and cytoplasmic protein methylation (Tier 3–4 functions) are severely compromised.

## 6. Epigenetic Architecture and Brain Methylation Patterns in ASD

A key feature of autism is the distinct regional pattern of epigenetic dysregulation [127]. The prefrontal and temporal cortices, crucial for social and language functions, are severely impacted, whereas the cerebellum, responsible for motor control, largely remains unaffected. This pattern strongly supports the view that epigenetic dysregulation represents one important mechanistic layer within ASD, particularly in metabolically defined subgroups. If autism was caused by a broad metabolic failure or widespread chromatin disorganization, we would see uniform dysregulation throughout all brain regions. Instead, the pattern aligns with epigenetic vulnerability, particularly in cortical association areas—regions where gene expression is highly dynamically regulated after birth through activity-dependent epigenetic changes [128,129].

These regions rely on ongoing Tier 2–3 methyltransferase activity to keep social and language genes active during experience-driven circuit refinement. When SAM levels drop, this dynamic regulation is hindered. In contrast, cerebellar motor circuits are mainly governed by genes that are steadily expressed through Tier 1 mechanisms, which remain unaffected during SAM depletion.

The pattern of epigenetic dysregulation across regions is not random; it mirrors the principles of methyltransferase hierarchy and the unique developmental needs of different brain areas. This regional specificity strongly supports the idea that epigenetic dysregulation—rather than widespread metabolic issues—is the one potential mechanistic pathway contributing to ASD in specific contexts.

### 6.1. Comprehensive Epigenetic Landscape Beyond DNA Methylation

The epigenetic framework of autism spectrum disorder extends beyond DNA methylation to include a complex network of chromatin modifications that collectively influence gene expression during neurodevelopment. Large-scale genetic research has identified that key genes disrupted in autism are involved in chromatin regulation, particularly those mediating histone methylation and demethylation, highlighting the centrality of epigenetic dysfunction. These modifications operate through coordinated networks involving HMTs, HDMs, and DNMTs, which work together to establish region-specific gene expression patterns essential for cortical development, synaptic formation, and social cognition.

Epigenetic dysregulation in ASD involves several interconnected mechanisms, including abnormal DNA methylation patterns (described earlier), changes in histone modifications (notably H3K4me2, H3K9me2, and H3K27me3), disrupted chromatin remodeling complexes, and impaired communication between DNA methylation and histone modifications [92,94,112]. This complex epigenetic disruption leads to lasting yet potentially reversible changes in gene expression that extend from development into adulthood, highlighting opportunities for therapies targeting epigenetic processes.

### 6.2. Regional Brain Methylation and Histone Modification Patterns

During brain development, DNA methylation and histone modifications show notable regional specificity in ASD, with distinct patterns associated with specific clinical features. Available studies suggest that various brain areas exhibit epigenetic differences affecting both DNA and histone marks (Table 5), but the evidence is heterogeneous across species, tissues, assays, and study designs, and some entries are best regarded as integrative summaries rather than uniformly replicated human findings.

The prefrontal cortex (PFC) exhibits significant epigenetic dysregulation, including widespread DNA hypomethylation (15–20%), a substantial reduction in H3K4me2 levels (−25%), and unexpectedly increased repressive H3K9me2 marks (+38%) [94,133]. This combination—loss of activating marks and gain of repressive marks—creates a “locked” chromatin state that hampers the expression of genes vital for social cognition (RELN, OXTR, SHANK3) and synaptic plasticity (EGR1, ARC) [94,133]. Post-mortem analysis of PFC tissue (Brodmann Area 9) from individuals with autism reveals a unique epigenetic pattern compared to age- and sex-matched controls, with the extent of H3K4me2 reduction correlating with the severity of social impairments [104,111,134,135].

The temporal cortex experiences notable epigenetic disruption, primarily impacting genes linked to language, such as CNTNAP2, NRXN1, and NLGN3. This disruption includes decreased H3K4me2 and increased H3K9me2, which collectively suppress synaptic adhesion molecules essential for auditory processing and language understanding [94,133]. In contrast, the cerebellum shows minimal epigenetic alterations and preserves motor functions, suggesting that motor and cerebellar systems are more resistant to epigenetic dysregulation than cortical social and language networks. This difference is key to understanding the ASD phenotype, characterized by social and communication difficulties but relatively preserved motor skills [7,124].

### 6.3. LSD1/KDM1A: The H3K4me2 Demethylase as a Therapeutic Target

Lysine-specific demethylase 1 (LSD1, also called KDM1A) has become a key epigenetic regulator; it is disrupted in autism and a promising but still largely preclinical therapeutic target. LSD1 is a FAD-dependent amine oxidase that removes methyl groups from mono- and dimethylated H3K4 (H3K4me1/2), and sometimes H3K9me1/2 in certain situations. It functions in several repressor complexes, including CoREST, NuRD, and CtBP, where it helps silence genes by demethylating histones and recruiting other chromatin modifiers [136,137,138].

Evidence consistently indicates abnormal LSD1 function in autism. Mutations in the LSD1 gene (KDM1A) have been identified in individuals with intellectual disability and autism-like traits, including three pathogenic mutations (Glu403Lys, Asp580Gly, Tyr785His) affecting active-site residues and partially impairing enzyme activity [110,139]. Additionally, LSD1 expression and activity are disrupted in autism mouse models: Shank3-deficient mice show altered LSD1 complex composition and increased H3K4me2 demethylation activity in the PFC, resulting in a 25% reduction in H3K4me2 levels compared to wild-type. Human post-mortem studies also support these findings, revealing significantly lower H3K4me2 levels in the PFC (Brodmann Area 9) among individuals with autism, with this reduction correlating with the severity of social impairments (r = −0.68, *p* < 0.001) [104,112].

The functional consequences of LSD1-mediated H3K4me2 depletion are profound and autism-relevant [140]: LSD1-mediated H3K4me2 depletion is associated with reduced expression of social cognition genes (e.g., EGR1), altered NMDA receptor subunit expression (GRIN2A/GRIN2B), and impaired activity-dependent synaptic plasticity. In preclinical models, pharmacological LSD1 inhibition restores H3K4me2 at these loci, promotes chromatin decompaction, and reactivates the associated transcriptional programs, with reported behavioral improvement even when treatment is initiated in adulthood; these findings remain preclinical and have not been replicated in human ASD cohorts [94].

#### GSK-LSD1

GSK-LSD1, an irreversible LSD1 inhibitor, is the most extensively studied compound in this class and has been reported in preclinical ASD models to reduce repetitive behaviors, increase social interaction time, normalize NMDAR-mediated excitatory postsynaptic currents in prefrontal cortex, and restore H3K4me2 and associated gene expression, with effects persisting after treatment cessation. TAK-418, a second-generation LSD1 inhibitor with improved CNS penetration, shows similarly targeted therapeutic effects in preclinical models without altering wild-type behavior [94,135].

### 6.4. G9a/EHMT2: The H3K9me2 the Methyltransferase and Paradoxical Therapeutic Target

Euchromatic histone methyltransferase 2 (EHMT2), also called G9a, adds mono- and dimethyl groups to histone H3 at lysine 9 (H3K9me1/2), creating a chromatin mark linked to gene silencing [133,141]. G9a mainly works as part of a heterodimer with its paralog EHMT1/GLP (G9a-like protein), which accounts for most of the H3K9me2 in euchromatic regions across the genome. Unlike H3K9me3, which signals stable heterochromatin, the H3K9me2 set by G9a/GLP influences temporary gene silencing in active chromatin during development, especially playing a key role in neurodevelopment [133].

G9a is abnormally upregulated in ASD, contrasting with LSD1 dysregulation which leads to a loss of activating H3K4me2 marks. In autism, this results in an excessive build-up of repressive H3K9me2 marks, [133,142,143] effectively creating a “double lock” on gene expression. Genes crucial for social behavior and synaptic function are affected: they lose activating H3K4me2 marks and simultaneously gain repressive H3K9me2 marks, leading to significant silencing.

Several studies show G9a dysregulation in both autism models and human patients. In Shank3+/ΔC and related ASD mouse models, prefrontal cortex EHMT2/G9a protein and H3K9me2 levels are elevated relative to wild-type controls, with ChIP-seq data showing H3K9me2 enrichment at promoters of autism-relevant synaptic genes during key postnatal developmental windows; proposed drivers of this G9a overactivity include compensatory upregulation in response to reduced SAM availability, as described in the tiered hierarchy model, with downstream transcriptional repression of synaptic and social cognition genes. The G9a inhibitor UNC0642 reverses stress-induced social and anxiety-like deficits in preclinical models and shows broader neuroprotective effects across other neurological disease models, with reported selectivity for rescuing deficits in disease models without altering wild-type behavior; as with LSD1 inhibitors, these findings remain preclinical and are not yet supported by human ASD trial data [110,143].

Consistent evidence shows that H3K9me2 dysregulation plays a key role in autism, with higher levels in the PFC, links to social deficits, and therapeutic effects from G9a inhibition. This histone modification acts as a “molecular integrator,” converting various genetic and environmental risks, such as Shank3 mutations, inflammation, and developmental stress, into a shared epigenetic pattern marked by the suppression of genes involved in social behavior.

## 7. Conclusions

### 7.1. The Epigenetic Framework of ASD and Its Therapeutic Potential

Autism arises from coordinated epigenetic dysregulation, in which reduced activating marks (DNA methylation and H3K4me2) coexist with excessive repressive marks (H3K9me2 and H3K27me3) in key cortical regions. This creates a functional “double lock” that selectively silences genes governing synaptic connectivity, neurotransmission, and social behavior [140,144].

**Figure 3:**
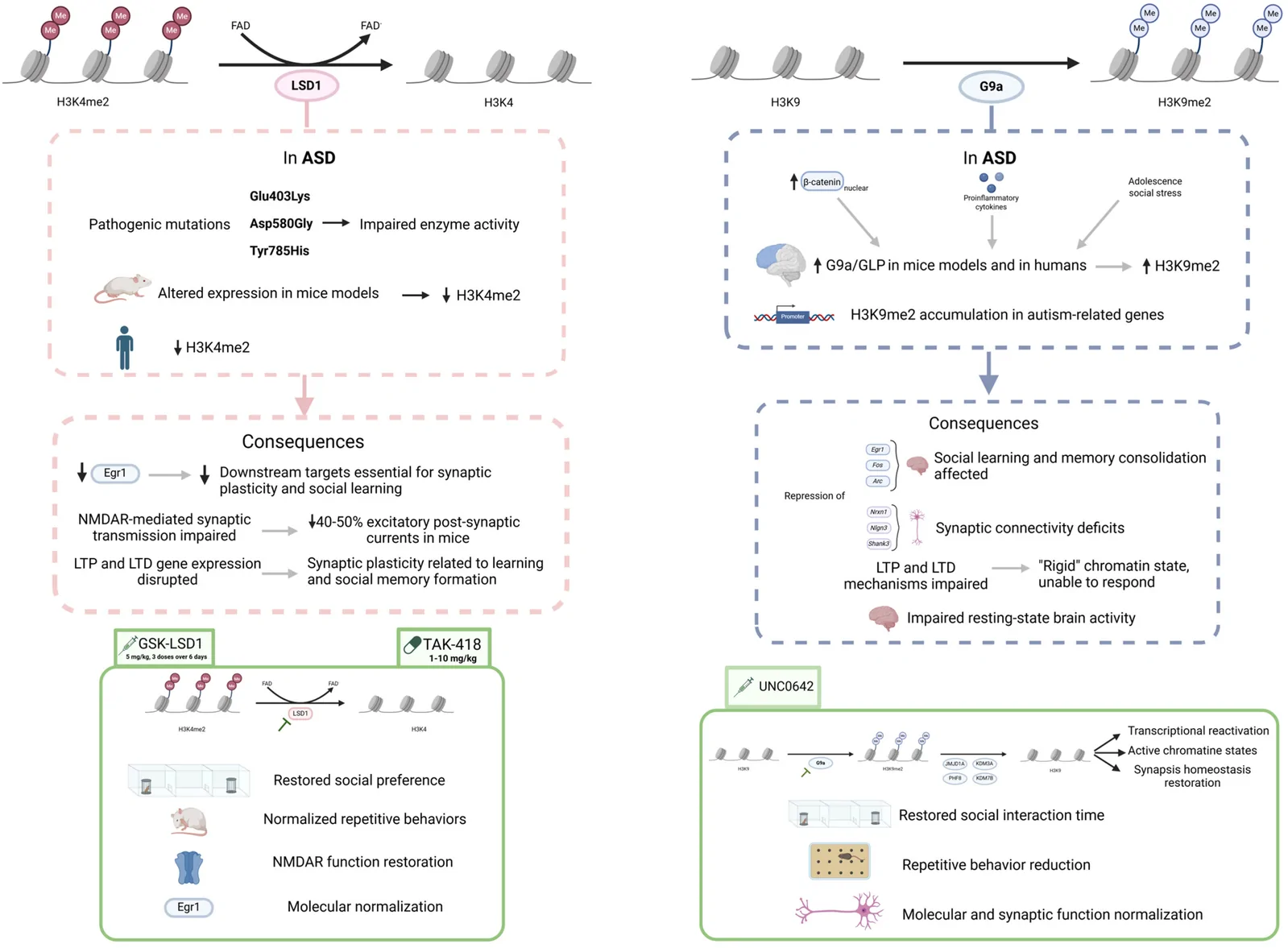
Epigenetic modifications mediated by LSD1 and G9a, their dysregulation in ASD, and the resulting molecular and functional consequences. Pharmacological inhibition of LSD1 using GSK-LSD1 and TAK-418, and of G9a using UNC0642, restores epigenetic balance and rescues ASD-related phenotypes in mouse models.

This dysregulation is regionally and temporally specific, with the prefrontal and temporal cortices being most affected during postnatal critical periods of circuit refinement. The targeting of H3K9me2 to autism-relevant genes, including SHANK3, NRXN1, NLGN3, NMDAR, and GABA receptor genes, demonstrates that epigenetic repression is not random but is functionally organized around the ASD phenotype. Crucially, these chromatin abnormalities are reversible. Pharmacological inhibition of G9a (EHMT2) and LSD1 partially normalizes histone balance, reactivates silenced synaptic genes, and rescues social and cognitive behaviors, even when treatment is initiated in adulthood. This shows that ASD is not a fixed epigenetic scar but a modifiable chromatin state (Figure 3).

Together, these findings position the epigenetic machinery as the final common pathway linking genetic vulnerability, folate–SAM metabolism, inflammation, and neurodevelopmental timing. Targeting chromatin regulators, therefore, offers a rational, potentially disease-modifying strategy for autism across the lifespan.

### 7.2. Folate Receptor Autoantibodies and Altered Folate Transport

FRAA seroprevalence estimates in ASD vary substantially across studies, ranging from roughly one-quarter to over 70% of children in some cohorts, depending on assay methodology, population, and control selection; not all studies confirm an elevated FRAA prevalence in ASD relative to controls (e.g., Phunsawat et al., 2022 [145], reported no significant FRAA association in their cohort). The 71–75% figure discussed below derives from a specific published cohort and should not be generalized as the prevalence across all ASD populations; it is presented here to characterize a clinically relevant FRAA-positive subgroup rather than as an ASD-wide estimate. This subgroup develops FRAA, which block folate transport across the blood–brain barrier and compromise CNS folate homeostasis (Table 6). The development of FRAAs greatly amplifies the ASD phenotype and identifies a mechanistic subgroup of ASD that can be targeted with folate replacement therapy. Children with high-titer FRAAs (>35–40% of the FRAA+ group) experience the most severe symptoms, such as developmental regression and seizures. Notably, FRAA+ children respond very well to folinic acid treatment, with 78–85% showing significant improvements within 2–5 weeks. This indicates that FRAA-related folate deficiency is a treatable component of ASD’s underlying mechanisms [93,146,147].

### 7.3. Toward a New Era: Epigenetic Medicine for Autism

The convergence of biomarker-driven precision medicine with the mechanistic understanding of autism pathophysiology marks an important shift in therapeutic thinking. Rather than applying uniform treatments across heterogeneous populations, future interventions will be dynamically calibrated to individual biological signatures, ensuring that therapies evolve alongside our expanding knowledge of each patient’s specific epigenetic and metabolic perturbations. This personalization paradigm reflects the biological reality that autism is not a monolithic disorder but rather a spectrum of distinct etiological pathways that converge on shared phenotypic outcomes. The evidence supporting folate-based rescue therapies, rigorously validated through clinical translation and mechanistic investigation, establishes a critical foundation for more sophisticated interventions. Folate supplementation addresses a fundamental metabolic node, the one-carbon cycle, that impinges on multiple levels of cellular dysfunction in autism, from DNA methylation capacity to antioxidant defense. This mechanistic centrality suggests that folate-based approaches will remain a cornerstone of autism therapeutics, potentially enhanced by personalized optimization informed by individual genotypes and biomarker profiles.

Beyond these foundational interventions, the emerging landscape of epigenetic therapeutics targeting G9a/EHMT2, LSD1/KDM1A, and HDAC6 represents a potential future direction for treating ASD at a mechanistic level. Rather than treating downstream symptoms, these agents directly rebalance chromatin states and correct SAM allocation dysfunction, addressing the fundamental cellular processes that drive autism-related transcriptional dysregulation. The capacity of these epigenetic modifiers to rescue behavioral, synaptic, and molecular phenotypes in preclinical models demonstrates that the underlying epigenetic architecture of autism is not immutable but rather amenable to pharmacological correction even in established disease states.

As clinical development and preclinical research advance, therapeutic strategies are becoming more complex and integrated. Personalized folate supplementation, guided by *MTHFR* and MTRR genotyping in combination with plasma and cerebrospinal fluid biomarkers, will allow clinicians to tailor dosing and formulations to individual metabolic capacities. Screening for FRAAs will help identify autoimmune subgroups of autism that require specific immunological treatments and targeted folinic acid therapy, distinguishing this mechanistically unique group from the broader ASD spectrum. Next-generation epigenetic therapies targeting both LSD1 and G9a pathways, known as dual-target approaches, aim to leverage the “double lock” mechanism of autism-related epigenetic dysregulation. These agents can simultaneously restore activating histone marks and reduce repressive modifications, potentially achieving more comprehensive normalization of gene expression than single-target strategies. Adding HDAC6 inhibitors to LSD1 and G9a targeting in triple-target approaches will broaden therapeutic potential by addressing acetylation-dependent processes involved in protein homeostasis and cellular stress, leading to a more complete epigenetic rebalancing.

Interventions will focus on specific compartments to regulate SAM dysregulation and spatial distribution issues in the one-carbon cycle. Mitochondrial-targeted folate derivatives and agents that replenish mitochondrial and cytoplasmic SAM pools extend beyond traditional supplements, offering precise subcellular therapy. These methods could be particularly advantageous for autism cases with confirmed mitochondrial dysfunction or energy deficits. Integrating microbiome modulation can boost folate absorption and synthesis, acknowledging the gut microbiota’s vital role in folate bioavailability and metabolism. Using prebiotics or probiotics to increase folate-producing bacteria, along with diet optimization and targeted antimicrobials against dysbiotic populations, may enhance the efficacy of folate supplements and reduce required doses. Anti-inflammatory strategies targeting neuroinflammation and immune dysregulation will address the inflammatory environment that sustains epigenetic and transcriptional dysregulation in autism. Approaches such as microglial modulation, cytokine antagonism, and complement inhibition might work synergistically with epigenetic therapies to foster a conducive environment for transcriptional stability. Likewise, antioxidant strategies aimed at restoring glutathione redox balance will counteract oxidative stress-related epigenetic alterations and support folate and SAM-dependent antioxidant systems. Mitochondrial therapies, including CoQ10 analogs, carnitine supplements, and new agents targeting the electron transport chain, aim to enhance bioenergetic capacity and overcome metabolic limitations that impede one-carbon cycle flux and epigenetic enzyme activity. By focusing on the energy foundation of epigenetic remodeling, these strategies could improve the efficacy of epigenetic treatments, especially in metabolically impacted autism groups. mTOR pathway modulators are likely to serve as valuable adjuncts for autism cases with documented dysregulation of this nutrient-sensing pathway. Carefully calibrated mTOR inhibition, which preserves essential anabolic processes for synapse development while reducing abnormal signaling, may augment other interventions and address autism-related issues like seizures and metabolic problems. Stratified clinical trials employing methylation biomarkers, genetic polymorphism profiles, and emerging epigenomic signatures are set to become the standard for developing treatments for autism. Instead of enrolling heterogeneous groups and settling for modest effects, these trials will target patients grouped by specific molecular subtypes most likely to benefit from targeted therapies. This approach will accelerate clinical translation, improve statistical power, and ultimately identify the best patient populations for each therapy.

The merging of folate biology with epigenetic mechanisms is more than just a technical step in autism neuropharmacology; it illustrates the most transformative aspects of translational neuroscience. When molecular insights, clinical precision, and therapeutic innovation come together, genuine hope shines for families affected by autism. This combined approach shifts the focus from merely managing symptoms to actively modifying the disease through mechanistic understanding. The future of neurodevelopmental medicine increasingly relies on the precision, integration, and clarity of mechanisms. It envisions personalized treatments tailored to individual biology, replacing generic methods and radically improving outcomes for those on the autism spectrum.

### 7.4. Conclusions

This review suggests that disruptions in folate-dependent one-carbon metabolism and imbalances in SAM/SAH might increase epigenetic vulnerability in certain biologically defined subgroups of autism spectrum disorder (ASD). The proposed SAM-centered hierarchy offers a conceptual model connecting metabolic dysfunction with specific changes in DNA and histone methylation, involving pathways like LSD1/KDM1A and G9a/EHMT1/2. However, this model remains hypothetical rather than a confirmed mechanism across the diverse ASD population. Current clinical evidence mainly supports folinic acid therapy for particular subgroups, especially those with folate receptor alpha autoantibodies or cerebral folate deficiency, while drugs targeting LSD1 and G9a are still mainly in preclinical development. Future research should test this hierarchy in disease-relevant neuronal models and include genetic, metabolic, and epigenetic biomarkers in well-stratified clinical trials. Such efforts may help determine whether modulating folate–SAM metabolism and chromatin regulation leads to more precise, biologically based treatments for specific ASD subgroups.

## Figures and Tables

**Table 1:** Genetic variants in folate metabolism and their reported relationships with ASD risk, metabolic effects, and epigenetic implications. Values should be interpreted in the context of study design, population, and nutritional environment; odds ratios are not interchangeable across cohorts, and mechanistic inferences are stronger for some variants than for others. ↓ = low/decreased; ↑ = high/increased.

Gene	Polymorphism	ASD Risk OR	Mechanism	Epigenetic Impact	Clinical Features	References
*MTHFR*	C677T	1.89 (no fortification)	60% enzyme activity reduction (homozygotes)	Global DNA hypomethylation (−15–25%), H3K4me3 ↓, H3K9me2 ↑	Social deficits, language delay, seizures, regression	[50,81,82,102]
*MTHFR*	A1298C	1.25–1.35	35–60% activity reduction	Moderate methylation dysregulation	Mild developmental delay	[50,82,86]
*MTHFR*	C677T/A1298C	2.15 (compound het)	65–75% combined activity reduction	Severe hypomethylation	Severe phenotype, responsive to folinic acid	[86]
MTR	A2756G	1.45	Methionine synthase activity reduction	SAM synthesis impairment	Augments *MTHFR* effects	[29,102]
MTRR	A66G	1.32–2.01	MS reductase cofactor cycling impairment	B12 methylation capacity ↓	Language improvement with folinic acid	[102]
FOLR1	G80A	1.67	Cerebral folate transport deficiency	Prenatal programming dysfunction	Regression, seizures, autism features	[55,56,57]
DHFR	Del19bp	2.69	DHFR overexpression, unmetabolized FA	Impaired neural methylation	Cognitive deficits	[61,62]
RFC1	A80G	1.41	Reduced folate transport efficiency	Tissue folate availability ↓	Neurodevelopmental delay	[52,53,54]

**Table 2:** Literature-based profiles of SAM, SAH, and methylation status in ASD-related subgroups and comparison populations. Unless otherwise specified in the cited studies, these entries should be treated as descriptive summaries rather than harmonized pooled estimates; subgroup values may derive from different cohorts, analytical platforms, and case definitions. For subgroup entries labeled as integrative estimates, values are presented as approximate ranges rather than fixed decimals with standard deviations, and each row should be cross-referenced to its specific source study rather than treated as a single harmonized measurement.

Study Population	SAM (ng/mL)	SAH (ng/mL)	SAM/SAH Ratio	Statistical Comparison	Methylation-Related Interpretation	References
Autistic disorder, predominantly regressive onset (*n* = 20; 19/20 with regression)	75.8 ± 16.2	28.9 ± 7.2	2.9 ± 0.8	SAM: *p* < 0.01; SAH and ratio: *p* < 0.001 vs. controls	Reduced peripheral methylation potential	[77]
Typically developing controls (*n* = 33)	96.9 ± 12.0	19.4 ± 3.4	5.2 ± 1.3	Reference group	Reference profile	[77]
Autistic disorder (*n* = 80)	84.3 ± 11.0	23.3 ± 7.9	4.0 ± 1.7	All three comparisons: *p* < 0.0001 vs. controls	Reduced peripheral methylation potential	[120]
Typically developing controls (*n* = 73)	93.8 ± 18.0	18.8 ± 4.5	5.5 ± 2.8	Reference group	Reference profile	[120]
Autistic disorder, sibling-paired subset (*n* = 40)	61.6 ± 8.9	20.0 ± 4.6	3.29 ± 1.10	Cases vs. siblings: SAM *p* < 0.006; SAH and ratio *p* < 0.001; cases vs. unrelated controls *p* < 0.001	Reduced methylation potential; global DNA methylation 3.03 ± 0.80% 5 mC	[92]
Unaffected paired siblings (*n* = 40)	70.7 ± 19.4	16.9 ± 3.9	4.4 ± 1.7	Paired comparison group	Global DNA methylation 3.9 ± 0.7% 5 mC	[92]
Unrelated typically developing controls (*n* = 54)	71.0 ± 15.6	14.8 ± 4.1	5.08 ± 1.80	Reference group	Global DNA methylation 4.13 ± 1.0% 5 mC	[121]
Children with ASD (*n* = 59)	83.2 ± 13.7	20.8 (18.1–24.5) ᵃ	3.87 ± 0.93	SAM and SAH: *p* < 0.001; ratio: *p* = 0.0001 vs. controls	Higher peripheral SAM/SAH ratio; direction differs from earlier cohorts	[121]
Neurotypical children (*n* = 40)	66.2 ± 14.9	37.9 (29.2–40.8) ᵃ	2.00 ± 0.76	Reference group	Reference profile for this cohort	[121]
ASD vs. controls, random-effects meta-analysis	Hedges’ g = −0.60 (95% CI −0.86 to −0.34)	Hedges’ g = +0.69 (95% CI 0.43–0.94)	Hedges’ g = −0.98 (95% CI −1.30 to −0.66)	Eight studies for each SAM-related outcome	Overall evidence of lower peripheral methylation potential; not an absolute concentration estimate	[109]

ᵃ Integrative estimate derived from cross-study subgroup analyses; no single published cohort directly measures all columns for this subgroup. Values should be interpreted as approximate summaries rather than harmonized pooled data.

**Table 3:** Proposed sensitivity of selected methyltransferases to SAM limitation and folate-related metabolic stress. Km ranges derive from enzymology and biochemical literature where available; cellular abundance and ASD impact are inferential or context-dependent for several enzymes, and the tier system is presented here as a hypothesis-generating framework rather than an established ASD-specific hierarchy.

Enzyme	Tier	Km SAM (μM)	Cellular Abundance	ASD Impact	Folate Sensitivity	References
DNMT1	1	0.5–2.0	High	Essential DNA maintenance	Low	[47,122]
PRMT1	1	1.0–3.0	Very High	Essential protein functions	Low	[47,123]
tRNA Methyltransferases	1	0.8–1.5	Moderate	Protein synthesis preserved	Low	[47,69]
DNMT3A	2	1.5–3.0	Moderate	Early decline, hypomethylation	Moderate	[47,69]
EZH2	2	0.5–1.5	Variable	Context-dependent effects	High	[47,69]
SETD1A	2	1.0–2.0	Moderate	Variable priority	High	[47,69]
G9a/EHMT2	3	0.8–2.5	High in neurons	Upregulated in ASD	High	[110,124,125]
PRMT5	3	1.5–4.0	Moderate	Myelination effects	High	[47,69]
DNMT3B	3	2.0–4.0	Low (adult)	Early embryonic effects	High	[47,69]
PRMT7	4	2.0–5.0	Low	Vesicle cycling, ID features	Very High	[47,69]
PRMT8	4	1.5–3.5	Neuron-specific	Social behavior deficits	High	[47,69,126]

**Table 4:** Functional compartmentalization of SAM and candidate methylation pathways. Concentration ranges primarily reflect the biochemical and cell-biology literature rather than direct measurements in ASD-relevant human neurons; relevance to ASD is therefore inferential unless explicitly supported by disease-specific studies.

Compartment	SAM Conc. (μM)	Primary Functions	Key Enzymes	Protection Level	References
Nuclear	100–150	DNA/Histone methylation	DNMT1, SETD1A	Very High	[33,47,69]
Euchromatin	120–180	Active transcription	SETD1A, DNMT3A	Very High	[47,69]
Heterochromatin	80–120	Gene silencing	G9a, EZH2	High	[47,69]
Nucleolar	150–200	rRNA processing	FBL, NOP2	High	[47,69]
Cytoplasmic	50–100	Protein methylation	PRMT1, GNMT	High	[47,69]
Mitochondrial	20–50	Metabolite methylation	NNMT, PNMT	Moderate	[33,47]

**Table 5:** Epigenomic changes associated with brain regions in ASD and related model systems. The evidence varies, including human post-mortem studies, peripheral-to-brain inferences, animal models, or integrated estimates. Each row should be interpreted in light of the species, tissue source, assay, and study type. Entries for hippocampus, cerebellum, dorsal raphe, and caudate are primarily inferred from animal models or cross-condition extrapolations; no dedicated human ASD post-mortem ChIP studies for these regions have been identified. Numerical % changes outside the PFC/striatum rows should not be treated as directly replicated human ASD measurements.

Brain Region	Global DNA Methylation Change	H3K4me2 Status	H3K9me2 Status	H3K27me3 Status	Key Genes Affected	Functional Impact	Clinical Correlation	References
Prefrontal Cortex	−15 to −20%	Decreased (−25%)	Increased (+38%)	Minimal change	RELN, OXTR, SHANK3, EGR1	Social cognition, NMDAR function	Social deficits, impaired Theory of Mind	[90,94,110,130,131,132]
Temporal Cortex	−12 to −18%	Decreased (−18%)	Increased (+32%)	Increased (+15%)	CNTNAP2, NRXN1, NLGN3	Language processing, synaptic connectivity	Communication delays, language impairment	[94,128]
Hippocampus	−8 to −12%	Decreased (−12%)	Increased (+20%)	Increased (+10%)	BDNF, CREB1, ARC	Memory/learning, synaptic plasticity	Learning difficulties, memory deficits	[94,128]
Cerebellum	−5 to −8%	Minimal change	Minimal change	Minimal change	Minimal changes	Motor control	Preserved motor skills	[94,128]
Dorsal Raphe	−10 to −15%	Decreased (−15%)	Increased (+25%)	Increased (+12%)	HTR2C, RABGGTB, TPH2	Serotonergic function, sensory processing	Sensory issues, anxiety	[90,130,131]
Caudate Nucleus	−6 to −10%	Decreased (−10%)	Increased (+18%)	Moderate increase	DRD2, COMT, DAT1	Executive function, reward processing	Attention problems, repetitive behaviors	[90,130,131]
Striatum	−8 to −14%	Decreased (−20%)	Increased (+30%)	Increased (+10%)	DRD1, DRD2, DARPP32	Motor control, habit formation	Repetitive behaviors, motor stereotypies	[90,130,131]

**Table 6:** Folate receptor alpha autoantibodies, cerebrospinal folate findings, and folinic acid treatment responses across clinical subgroups. Response rates and timelines vary by subgroup, trial design, and outcome measure and should not be generalized to unselected ASD populations.

Population	FRAA Prevalence (%)	High-Titer FRAA (%)	CSF Folate Reduction	Treatment Response	Improvement Timeline	References
ASD Children	71–75	25–30	15–25%	78%	2–5 weeks	[93,146,147]
Typically Developing	<5	<1	Normal	N/A	N/A	[146,147]
ASD + Language Delay	85–90	35–40	25–35%	85%	2–4 weeks	[93,147]
ASD + Seizures	80–85	30–35	20–30%	82%	3–6 weeks	[93,147]
ASD + Regression	75–80	28–32	18–28%	80%	2–5 weeks	[93,147]
ASD + GI Issues	70–75	22–28	12–22%	75%	3–7 weeks	[146,147]

## Data Availability

No new data were created or analyzed in this study. Data sharing is not applicable to this article.
